# The Effect of Considering Future Consequences on College Students’ Perceptions of Stress in Relation to Resilience and Sense of Meaning in Life

**DOI:** 10.3390/bs15030258

**Published:** 2025-02-23

**Authors:** Nanbo Wang, Ge Xu, Song Zhou, Lixia Jiang, Qingli Guan, Man Leng

**Affiliations:** 1 Department of Psychology, School of Health, Fujian Medical University, Fuzhou 350122, China; 2 School of Psychology, Fujian Normal University, Fuzhou 350117, China; qsx20230503@student.fjnu.edu.cn (G.X.);

**Keywords:** HAPA model, perceived stress, resilience, meaning in life, consideration of future consequence

## Abstract

The present study examines the moderated mediation model of resilience and meaning in life (MIL) within the Health Action Process Approach (HAPA) framework. A sample of 971 Chinese college students (mean age = 19.95; 69.5% female) completed measures of consideration of future consequences (CFCs), resilience, MIL, and perceived stress. The results supported the hypothesized model: CFCs negatively predicted perceived stress, and this relationship was partially mediated by resilience. MIL moderated the association between CFCs and perceived stress, with the search for meaning subdimension amplifying the negative relationship. In contrast, the presence of meaning did not exhibit a significant moderating effect. High levels of CFCs were significantly linked to lower perceived stress when the level of search for meaning was high. These findings highlight the dynamic interplay of cognitive and motivational factors in stress management, underscoring the potential of fostering resilience and meaning-seeking behaviors to promote well-being among college students.

## 1. Introduction

The search for meaning in an absurd world is itself the meaning.

----Albert Camus (1955)

This profound observation encapsulates the essence of human resilience and purpose, even in the face of challenges. College students worldwide are experiencing rising levels of perceived stress, a condition exacerbated by the rapidly accelerating pace of life and mounting societal demands. Recent research highlights the concerning prevalence of moderate to severe stress among this demographic. For instance, in the United States, stress rates have reached 58% (Srinivasa, 2022); in Iran, 92.4% (Ganjoo et al., 2021); and there is a significant upward trend in China (Gong et al., 2023). These findings reveal a global crisis that transcends cultural boundaries, fueled by cognitive, emotional, and motivational factors.

Previous studies have consistently shown that high levels of perceived stress have a detrimental effect on mental health, contributing to conditions such as depression, anxiety, and even physical pain (Litam et al., 2021). College students, who are undergoing a critical transition from adolescence to early adulthood, are especially vulnerable to heightened stress levels. Long-term exposure to persistent stress not only hampers academic performance (Bruffaerts et al., 2019) but can also lead to significant mental health challenges (de Girolamo et al., 2022). Given the negative consequences of prolonged stress, it is crucial to address perceived stress among college students to promote their well-being.

One effective theoretical framework for managing stress is the Health Action Process Approach (HAPA), which emphasizes two phases of behavior change: the pre-intentional motivational phase and the post-intentional volitional phase (Cao et al., 2023). In the motivational phase, cognitive components influence the emergence of the intent to change behavior. In the volitional phase, two key factors contribute to behavior change: action planning and maintenance (Malik et al., 2022). Specifically, HAPA underscores the importance of considering future consequences when making decisions about health-related behaviors, offering a novel perspective on stress management. Previous applications of the HAPA model have demonstrated its utility in understanding behaviors related to perceived stress (Silva-Smith et al., 2024; Ulrich et al., 2024).

Building on this framework, the present study explores how the consideration of future consequences (CFCs) influences stress perceptions, examining the mediating role of resilience and the moderating influence of meaning in life. Anchored in the Health Action Process Approach (HAPA) model, this research aims to uncover pathways for fostering well-being through a deeper understanding of these dynamics.

### 1.1. The Relationship Between Consideration for Future Consequences and Perceived Stress Among College Students

Perceived stress refers to an individual’s subjective evaluation of stressors and their perceived ability to cope with them (Liu et al., 2021). Among college students, stressors often stem from academic pressures, homesickness, and concerns about future career prospects (Gong et al., 2023). Lazarus and Folkman’s transactional theory of stress and coping suggests that stress arises when individuals perceive stressors as threats and assess their coping resources as insufficient (Wolfers & Schneider, 2021). Prolonged exposure to high levels of perceived stress can lead to a variety of negative outcomes, including fatigue, anxiety, depression, and more severe psychological disorders, such as suicidal ideation (Cohen et al., 2007; S. Zhou et al., 2021).

According to the HAPA model, stress management, which is beneficial for mental health, also unfolds in two phases. In the motivational phase, individuals recognize the importance of managing stress, assess the risks associated with chronic stress, and form intentions to adopt stress-reducing behaviors. However, translating these intentions into consistent and effective action requires progression into the volitional phase, where strategies are planned, executed, and maintained over time. Both phases are essential for successful stress management, as they emphasize the interplay between forming intentions and carrying out actions.

Within the HAPA framework, the concept of consideration of future consequences (CFCs) plays a pivotal role (Cao et al., 2023). CFC refers to the extent to which individuals consider future consequences when making decisions (Shen et al., 2023). Individuals who are more attuned to future consequences tend to prioritize long-term outcomes over immediate gratification (Shen et al., 2023). Those high in CFCs are more likely to think about the potential long-term consequences of chronic stress, such as its impact on physical health, mental well-being, and overall quality of life. Consequently, they are more inclined to focus on long-term solutions and develop detailed plans, which enhances their sense of control over the future and allows them to implement stress management strategies more effectively (Peetsma & Van Der Veen, 2011). Additionally, such individuals typically possess a strong sense of responsibility and self-discipline, enabling them to achieve their goals without distraction (D’Alessio et al., 2003). Their ability to plan for the future not only strengthens their emotional resilience but also helps them cope more effectively with challenging situations, especially during stressful times (Shen et al., 2023). For example, a study conducted during the COVID-19 pandemic found that individuals with higher levels of CFCs were able to shift their focus from immediate stress to future well-being, reducing anxiety and depression through the coping strategies they employed (Chen et al., 2021). Based on these findings, we hypothesize that CFCs serve as a key predictor of perceived stress; individuals with higher levels of CFCs tend to experience lower levels of perceived stress (H1).

### 1.2. The Mediating Role of Resilience

According to the stress–coping theory (Lazarus & Folkman, 1984), an individual’s perceived stress depends not only on their appraisal of a stressful event but also on their available resources and coping abilities. Resilience, as an important psychological resource for managing stress, may play a crucial mediating role in this process (Chen et al., 2021). Resilience refers to an individual’s capacity to adapt and thrive in the face of adversity, and it is widely regarded as a key personal resource for coping with stress (Connor & Davidson, 2003). Research has shown that resilience is effective in buffering the negative impact of external stressors, enabling individuals to cope with challenges more effectively (Çuhadar et al., 2023; Gong et al., 2023; Tao et al., 2023). Ong et al. (2006) found that individuals with high resilience exhibit reduced emotional reactivity to stressors, resulting in lower levels of perceived stress. Among college students, resilience has been shown to moderate stress, helping them navigate academic pressures and enhance overall well-being (Ye et al., 2020; Gong et al., 2023).

According to the HAPA model, behavioral change is the result of a multifactorial process in which motivational factors, such as consideration of future consequences (CFCs), influence behavioral outcomes like perceived stress through an individual’s internal resources (e.g., resilience). Research has shown that resilience is positively associated with CFCs (Seidmahmoodi et al., 2011). Due to their enhanced ability to plan for the future, individuals with high CFCs tend to invest more effort in acquiring the skills and resources necessary to achieve long-term goals (I.-J. Park & Jung, 2015) and proactively develop coping strategies. These efforts help them cultivate greater self-control and reduce impulsivity (Zimbardo & Boyd, 2015), thereby enhancing their self-regulation abilities and contributing to greater psychological resilience. When college students consider the future consequences of their decisions, this long-term thinking strengthens their resilience, making them less likely to break down and better able to maintain high levels of coping ability when facing stressors. Therefore, we propose that resilience may serve as a crucial mediator between consideration of future consequences (CFCs) and perceived stress (H2).

### 1.3. The Moderating Role of Meaning in Life

Meaning in life is defined as an individual’s experience of meaningfulness and their perception of having a clear sense of purpose or direction (Platsidou & Daniilidou, 2021). This sense of meaning is thought to emerge when individuals align their actions with long-term goals or desired future outcomes (Huang et al., 2023). According to Park’s meaning-making model, meaning in life significantly shapes an individual’s cognitive and emotional responses to stressors, helping them reinterpret challenges and reduce perceived stress (C. L. Park, 2010). For example, college students with a strong sense of meaning in life are more likely to view stressors as opportunities for growth rather than threats to their well-being. This perspective enables them to engage in more effective stress management strategies (Huang et al., 2023). In contrast, individuals with a weaker sense of meaning in life may experience heightened vulnerability to stress, reduced motivation, and greater susceptibility to negative emotional states, such as anxiety and depression (Guo et al., 2024). In severe cases, a lack of meaning in life can lead to serious psychological issues, including suicidal ideation, violence, and substance abuse (J.-J. Zhou et al., 2023).

Moreover, the effectiveness of considering future consequences in reducing perceived stress can vary from person to person. Specifically, for individuals with a strong sense of meaning in life, a greater consideration of future consequences is associated with lower perceived stress. These individuals are likely to use their future orientation to actively pursue meaningful goals (Huang et al., 2023), which can buffer against stress. In contrast, for those with a low sense of meaning in life, this relationship may be weaker or even reversed. According to the ego depletion (Baumeister et al., 1998), individuals with low meaning in life may experience heightened cognitive resource depletion when engaging in CFCs. This is because the absence of a coherent meaning framework forces them to allocate additional cognitive resources to manage existential uncertainty and emotional distress, leaving fewer resources available for effective future-oriented planning. As a result, these individuals may perceive future-related tasks as overwhelming and unmanageable, leading to increased stress levels. Furthermore, the lack of meaningful goals may prevent the “goal decoupling” effect proposed by Masicampo and Baumeister (2011), wherein concrete plans reduce the cognitive burden of unfulfilled goals. Without such decoupling, low-meaning individuals may experience persistent rumination about unresolved future concerns, further depleting their self-regulatory resources and amplifying stress. A heightened focus on future consequences could exacerbate stress, as these individuals may feel overwhelmed by their inability to connect their actions with a meaningful future. Therefore, we propose that meaning in life may moderate the relationship between consideration of future consequences and perceived stress (H3).

As posited by Steger et al. (2006), the concept of meaning in life is a multifaceted construct that encompasses both the presence of meaning (MIL-P) and the search for meaning (MIL-S) (Barnett et al., 2019). The presence of meaning refers to the actual experience of meaning in life, and there is general consensus that this experience is beneficial for subjective well-being (Lew et al., 2020). The search for meaning, on the other hand, refers to the desire and effort to find meaning in life or to improve one’s understanding of the meaning already experienced (Platsidou & Daniilidou, 2021). The presence of meaning is considered a trait-like protective factor against the impact of negative life events, including the COVID-19 pandemic (Baños et al., 2023), and is associated with higher levels of subjective well-being (Xu et al., 2022). However, the search for meaning has shown low positive correlations with life satisfaction, happiness, and positive affect (Platsidou & Daniilidou, 2021), and moderate to high correlations with anxiety and less effective use of coping resources (Elemo et al., 2024). When life is stable, the presence of meaning acts as a protective factor. However, during challenging life events, the search for meaning becomes more crucial (Elemo et al., 2024). Based on these inferences, we propose that the moderating effect of meaning in life is driven by the search for meaning, rather than the presence of meaning (H4).

**Figure 1:**
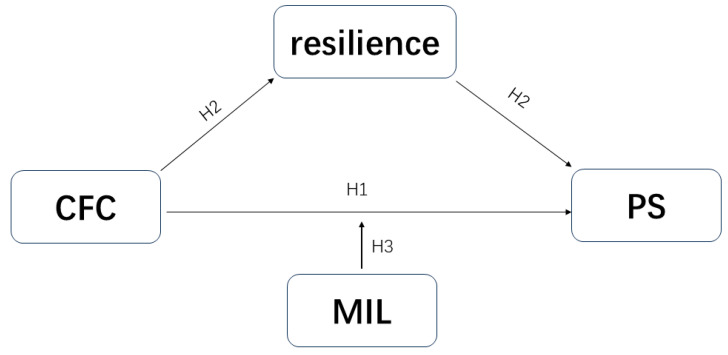
Hypothetical model. A hypothetical model of consideration of future consequences and perceived stress with resilience as the mediating variable and meaning in life as the moderating variable.

**Figure 2:**
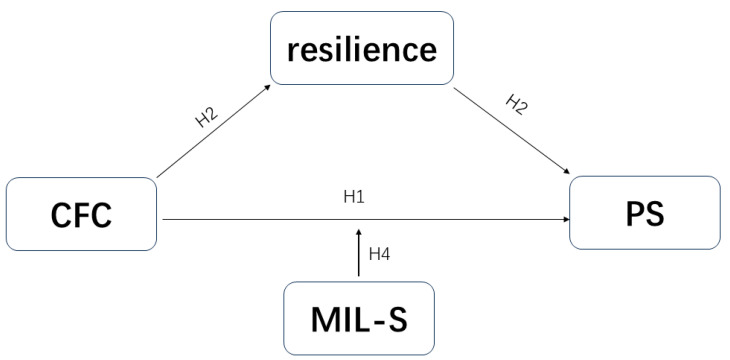
Hypothetical model. A hypothetical model of consideration of future consequences and perceived stress with resilience as the mediating variable and search for meaning as the moderating variable.

**Figure 3:**
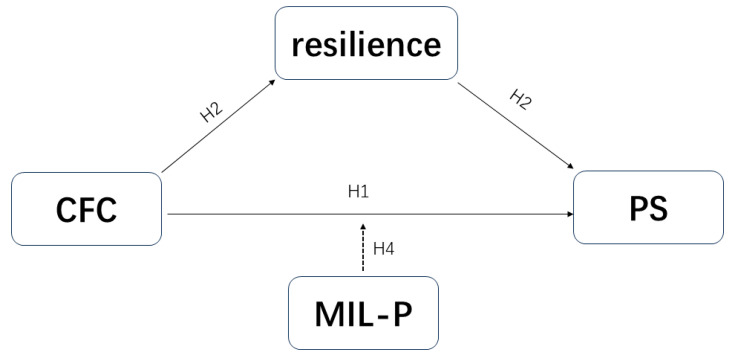
Hypothetical model. A hypothetical model of consideration of future consequences and perceived stress with resilience as the mediating variable and presence of meaning as the moderating variable.

Building on existing theoretical and empirical support, the present study aims to investigate the predictive role of consideration of future consequences in perceived stress among college students and to explore the underlying mechanisms through the mediating role of resilience and the moderating role of meaning in life. By constructing a moderated mediation model (see Figure 1, Figure 2 and Figure 3), this study will provide valuable empirical support for developing effective strategies to reduce perceived stress among college students.

## 2. Method

### 2.1. Participants

A total of 1020 participants were recruited through a Chinese online platform (https://www.wjx.cn/), URL (accessed on 12 September 2023) and offline paper questionnaires. After excluding the questionnaires with abnormal response patterns or those failed to pass the catch items, 971 valid questionnaires were finally obtained, with a recovery rate of 95.2%. Of the participants who submitted valid questionnaires, 296 (30.5%) were male and 675 (69.5%) were female, with a mean age of 19.95 years (*SD* = 1.90). All participants completed the questionnaire after understanding the purpose of the study and signing the informed consent form. The study was approved by the ethics committee of Fujian Normal University. And the data collection was carried out in accordance with the provisions of the World Medical Association Declaration of Helsinki.

### 2.2. Measures

#### 2.2.1. Perceived Stress

Perceived stress (PS) was measured using a two-item scale adapted from a previous study by Cohen et al. (1983). The items are as follows: “In the last month, how often have you felt that you were unable to control the important things in your life?” and “In the last month, how often have you felt difficulties were piling up so high that you could not overcome them?” This scale is rated on a 5-point scale, from 0 (never) to 4 (very often), with higher scores indicating higher levels of stress. The Cronbach’s alpha coefficient for this scale in the current study was 0.838. On one hand, longer questionnaires can lead to participant fatigue, resulting in dropout or careless responding. On the other hand, shorter questionnaires reduce the likelihood of defensive responses due to item redundancy (e.g., excessive questions about stress may provoke social desirability bias or response distortion) (S. Zhou et al., 2021). Moreover, the scale has been successfully applied before (Cohen et al., 1983; S. Zhou et al., 2021), supporting its applicability in the context of this study.

#### 2.2.2. Consideration of Future Consequence

Consideration of future consequences (CFCs) was measured using the consideration of future consequences scale, originally developed by Strathman et al. (1994). This scale consists of 12 items, such as, “I consider how things might be in the future and try to influence those things with my day-to-day behavior.” Items 3, 4, 5, 9, 10, 11, and 12 were reverse-scored. Responses to each item were rated on a 5-point Likert scale (1 = extremely uncharacteristic to 5 = extremely characteristic), with higher scores indicating higher levels of CFCs. The McDonald’s Omega value for this scale was 0.680.

#### 2.2.3. Resilience

Resilience was assessed using the 10-item Connor–Davidson Resilience Scale (Campbell-Sills & Stein, 2007) (e.g., “I can deal with whatever comes”). This version of the resilience scale has been validated among Chinese college students (Lyu et al., 2017). The scores are rated on a scale from 0 (completely untrue) to 4 (completely true), with higher scores indicating greater resilience. The McDonald’s Omega value for this scale was 0.898.

#### 2.2.4. Meaning in Life

The Meaning in Life Questionnaire was developed by Steger et al. (2006) and consists of 10 items, divided into two dimensions: *Presence of Meaning* (items 2, 4, 7, 8, 9), which measures the degree to which individuals feel their life is meaningful, emphasizing the outcome; and *Search for Meaning* (items 1, 3, 5, 6, and 10), which measures the degree of active pursuit of meaning in life, emphasizing the process. The questionnaire uses a 7-point Likert scale (1 = completely incorrect to 7 = completely correct), with item 2 being reverse-scored. Higher scores indicate a greater sense of meaning in life. The Chinese version of the questionnaire was revised by Wang and Dai (Yang et al., 2022), and it has demonstrated good reliability and validity. The McDonald’s Omega value for meaning in life, presence of meaning, and search for meaning are 0.812, 0.792, and 0.826, respectively.

### 2.3. Statistical Analysis

IBM SPSS 26.0 was used for descriptive and correlation analysis, while Model 4 and Model 5 of the PROCESS 4.0 macro program (http://www.afhayes.com, accessed on 2 October 2023) were employed to examine the mediating role of resilience and the moderating role of meaning in life between future consequences and perceived stress. Specifically, percentile bootstrapping, along with bias-corrected percentile bootstrapping with 5000 resamples, was used to construct 95% confidence intervals for the indirect effects.

## 3. Result

### 3.1. Descriptive Statistics and Correlation Analysis

The means, standard deviations, and correlation analyses of all variables are presented in Table 1. The results indicate that consideration of future consequences (CFCs) is significantly and negatively correlated with Perceived Stress (PS) (*r* = −0.144, *p* < 0.001). CFC is also significantly and positively correlated with resilience, meaning in life, presence of meaning, and search for meaning (*r* = 0.175, *p* < 0.001; *r* = 0.282, *p* < 0.001; *r* = 0.241, *p* < 0.001; *r* = 0.250, *p* < 0.001). Resilience, in turn, shows a significant negative correlation with PS (*r* = −0.316, *p* < 0.001). Additionally, resilience is significantly and positively correlated with meaning in life, presence of meaning, and search for meaning (*r* = 0.479, *p* < 0.001; *r* = 0.432, *p* < 0.001; *r* = 0.402, *p* < 0.001). Both meaning in life and presence of meaning are negatively correlated with PS (*r* = −0.141, *p* < 0.001; *r* = −0.187, *p* < 0.001). However, no significant correlation was found between search for meaning and PS. Furthermore, age shows a significant negative correlation with PS (*r* = −0.162, *p* < 0.001), while gender is significantly correlated with meaning in life (*r* = −0.054, *p* = 0.094).

### 3.2. Testing for Moderated Mediation Model

The results of the correlation analysis indicated that the relationships between consideration of future consequences, perceived stress, resilience, and meaning in life met the requirements for conducting a moderated mediation test. Notably, all predictors had variance inflation factors (VIFs) below 2, suggesting that there was no multicollinearity in the present study.

To test the mediating effects of resilience between consideration of future consequences and perceived stress, we used Model 4 of the PROCESS macro. The findings showed that consideration of future consequences negatively predicted perceived stress (B = −0.144, SE = 0.032, *p* < 0.001) (Table 2). This supports Hypothesis 1 (H1), indicating that consideration of future consequences significantly and negatively predicted perceived stress, even after controlling for gender and age.

Next, we conducted data analysis by introducing resilience as a mediating variable. The predictive effect of consideration of future consequences was weakened but remained significant and negative, while resilience was also a significant and negative predictor of perceived stress (*B* = −0.131, *SE* = 0.031, *p* = 0.003; *B* = −0.296, *SE* = 0.031, *p* < 0.001). These analyses indicated that consideration of future consequences positively predicted resilience (*B* = 0.175, *SE* = 0.032, *p* < 0.001), suggesting that resilience partially mediates the relationship between consideration of future consequences and perceived stress.

We then conducted a bootstrapping analysis with 5000 samples at 95% confidence intervals. The results indicated that resilience partially mediated the impact of consideration of future consequences, with an indirect effect value of −0.052 and a 95% confidence interval excluding 0 (*SE* = 0.013, [−0.148, −0.029], *p* < 0.001). The mediating effect accounted for 37.1% of the total effect. Accordingly, Hypothesis 2 (H2) was supported.

To test whether the mediation effect was moderated by meaning in life, we used Model 5 of the PROCESS macro and introduced an interaction term between meaning in life and consideration of future consequences. The results showed that the interaction term (meaning in life × consideration of future consequences) was a significant predictor of perceived stress (*B* = −0.062, *SE* = 0.026, *p* = 0.016) (Table 3), thus supporting Hypothesis 3 (H3).

To explore the interaction effect at different levels of the moderator, we conducted a simple slope test. The results showed that consideration of future consequences was strongly related to perceived stress at high levels (1 *SD* above the mean) of meaning in life (*B* = −0.150, *SE* = 0.038, *p* < 0.001). However, there was no significant statistical difference at low levels (1 *SD* below the mean) of meaning in life (*B* = −0.026, *SE* = 0.044, *p* = 0.548) (Table 4).

To further examine how the two factors of meaning in life influence perceived stress, we introduced the interaction of consideration of future consequences with the presence of meaning and the interaction of consideration of future consequences with the search for meaning into the model separately. The results revealed that the interaction of consideration of future consequences with the search for meaning was a significant predictor of perceived stress (*B* = −0.059, *SE* = 0.027, *p* = 0.027) (Table 5). However, the interaction of consideration of future consequences with the presence of meaning did not significantly influence perceived stress (*B* = −0.043, *SE* = 0.024, *p* = 0.075) (Table 6), thus supporting Hypothesis 4 (H4).

Additionally, we conducted a simple slope test to explore the interaction effect at different levels of the search for meaning. The results showed that consideration of future consequences was strongly related to perceived stress at high levels (1 *SD* above the mean) of the search for meaning (*B* = −0.158, *SE* = 0.038, *p* < 0.001). However, there was no significant statistical difference at low levels (1 *SD* below the mean) of the search for meaning (*B* = −0.039, *SE* = 0.045, *p* = 0.387) (Table 7).

## 4. Discussion

The period of higher education is a crucial phase in the lives of Chinese students. During this time, they face intense peer competition, high academic and research expectations, and a challenging job market. As a result, college students experience significant pressure. The negative effects of perceived stress may be more severe for college students compared to other demographic groups. This study focuses on college students and, based on the HAPA model, develops a moderated mediation model to explore how consideration of future consequences (CFCs), resilience, and meaning in life influence perceived stress. Specifically, we examine how resilience mediates the relationship between CFC and perceived stress and how meaning in life moderates the relationship between CFC and perceived stress. Our findings contribute to a deeper understanding of the mechanisms behind perceived stress, which could inform strategies to improve college students’ mental health.

The results show that CFC serves as a negative predictor of perceived stress, supporting Hypothesis 1. This finding aligns with the HAPA model, which suggests that individuals with high levels of CFC are more likely to engage in proactive planning and adaptive coping strategies, thereby shaping their stress perceptions. The impact of CFCs on perceived stress can be explained in several ways.

First, individuals with a high level of CFC are more likely to view stressors as manageable challenges rather than overwhelming threats. This is consistent with the motivational phase of the HAPA model, where a focus on long-term goals reduces the immediate emotional impact of stressors. For example, a student with a high CFC may perceive an upcoming exam not as a source of anxiety but as an opportunity to move closer to their academic goals, which mitigates perceived stress, this is in line with a recent study (Gao, 2023). Second, the volitional phase of the HAPA model emphasizes the role of action planning and coping strategies. High-CFC individuals tend to engage in proactive behaviors such as effective time management and problem-solving, which help them address potential stressors before they arise. By reducing uncertainty and exerting control over their environment, they experience lower stress levels. In contrast, low-CFC individuals may lack these proactive strategies, leaving them more vulnerable to being overwhelmed by immediate demands. Finally, the self-regulatory processes emphasized in the HAPA model help explain why high-CFC individuals are less prone to rumination and emotional reactivity. Their focus on long-term outcomes enables them to reframe negative experiences and maintain psychological resilience. This future-oriented perspective acts as a buffer against the adverse effects of stress, leading to a reduction in perceived stress.

The results of this study underscore the important role of resilience in mediating the relationship between CFCs and perceived stress, supporting Hypothesis 2. This finding also corroborates the theoretical framework of the HAPA model. In the HAPA model, CFC is part of the motivational phase, where individuals set goals and plan for the future. A high level of CFC leads people to anticipate stress and take proactive steps to manage it. However, the volitional phase, which involves executing these plans, requires resilience—the ability to adapt and recover from setbacks. Resilience acts as a critical mediator by enabling individuals to cope with stress and remain aligned with their long-term goals. While CFCs may drive individuals to plan and prepare for challenges, resilience allows them to effectively manage stress when it arises. Without resilience, even high-CFC individuals may struggle to cope with the demands they face, potentially increasing perceived stress. Specifically, individuals with high levels of CFCs tend to be more emotionally stable, better at resource allocation, and quicker to recover from negative stimuli—traits that predict their resilience. This suggests that individuals with high CFCs can enhance their resilience, ultimately leading to a reduction in perceived stress. By integrating resilience into the HAPA model, this study highlights its essential role in translating motivation into action, showing that resilience enables individuals to manage stress effectively and work toward long-term goals despite challenges. College students with strong CFCs may effectively influence the reappraisal of stressful events, thereby enhancing their resilience (Troy et al., 2023) and experiencing lower levels of perceived stress.

Moreover, the study reveals that meaning in life moderates the relationship between CFCs and perceived stress. Our findings indicate that meaning in life moderates this relationship, particularly through the search for meaning subdimension. Hypotheses 3 and 4 are supported. At high levels of the search for meaning (1 standard deviation above the mean), this dimension significantly strengthens the negative association between CFCs and perceived stress. This suggests that individuals actively seeking meaning in their lives are better able to leverage their future-oriented mindset to reduce stress, viewing stressors as challenges to be integrated into their long-term goals and personal growth. In contrast, at low levels of the search for meaning, the moderating effect is not significant. Individuals not actively seeking meaning may struggle to connect their future goals with present stressors, limiting their ability to reduce perceived stress even when they consider future consequences. This highlights the dynamic, motivational nature of meaning in life—particularly the role of meaning-seeking as an active, goal-directed process. However, the presence of meaning (a sense of meaning already established in life) did not significantly moderate the relationship between CFCs and perceived stress. This lack of moderation can be explained by the distinct psychological functions of “presence of meaning” and “search for meaning.” While, as we mentioned before, the presence of meaning reflects a stable, cognitive appraisal of life’s purpose, it may lack the motivational drive necessary to translate future-oriented thinking into actionable stress management strategies. According to the ego depletion (Baumeister et al., 1998), the presence of meaning, although providing a general sense of well-being, does not actively engage cognitive resources in goal-directed planning or problem-solving. In contrast, the search for meaning, as a dynamic and effortful process, mobilizes cognitive and emotional resources to align future goals with present actions, thereby enhancing the stress-buffering effects of CFCs. Additionally, the presence of meaning may function more as a passive buffer against stress rather than an active moderator. It provides a foundational sense of security and coherence but does not necessarily facilitate the adaptive reinterpretation of stressors that is crucial for reducing perceived stress in the context of CFCs. From the perspective of the HAPA model, this finding can be understood within the distinction between motivational and volitional phases. The presence of meaning represents a more stable, cognitive appraisal of life’s purpose, providing general life satisfaction and psychological well-being but not necessarily activating the dynamic, goal-directed processes that would help individuals effectively manage stress in response to CFCs.

In the HAPA model, the motivational phase emphasizes setting goals and forming intentions, while the volitional phase involves executing these goals through action plans and self-regulation. The search for meaning, which is an active and ongoing process, aligns with these phases by motivating individuals to seek and integrate meaning into their behaviors, thus enhancing their ability to cope with stress. The presence of meaning does not directly engage these volitional processes, which may explain why it does not significantly moderate the CFC–stress relationship. Rather, it reflects a stable sense of purpose that may not influence individuals’ active coping strategies in the same way as the search for meaning.

This study enhances our understanding of how resilience and meaning in life interact to influence the relationship between CFCs and perceived stress, extending the HAPA model. The HAPA model, which emphasizes motivation and volition in health behaviors, helps explain how CFCs shape stress responses through cognitive and behavioral processes. By incorporating resilience as a mediator, this research highlights the role of emotional and psychological resources in managing stress. Additionally, it shows how the search for meaning moderates the CFC-PS relationship, emphasizing that meaning is a dynamic process that influences stress perception. Practically, the findings suggest that fostering resilience and encouraging meaning-seeking behaviors can be effective strategies for stress management, particularly in academic and professional settings. Interventions that promote mindfulness, emotional regulation, and personal meaning-making can help individuals better cope with stress by aligning actions with long-term goals. In conclusion, this study provides valuable insights for both theoretical research and applied interventions to improve stress management and well-being.

It is important to acknowledge several limitations of this study, which should be addressed in future research. First, this study employed a convenience sampling methodology, which limits the ability to generalize the findings to a broader population. Specifically, the study participants were recruited from a single university, which may present certain institutional and regional characteristics that influence the relationships between consideration of future consequences (CFCs), resilience, meaning in life, and perceived stress. Students from this university may share similar socio-cultural backgrounds, educational experiences, and regional influences that could affect their psychological responses and coping mechanisms. Future studies should use random sampling to enhance the representativeness of the sample and improve the external validity of the findings. Another limitation is the imbalance in the gender distribution of participants. Given the nature of the constructs involved in our study (e.g., resilience, meaning in life, perceived stress), gender may influence how individuals respond to these variables. For example, women may report higher levels of perceived stress due to social factors, while men may underestimate stress due to cultural expectations of emotional restraint (Graves et al., 2021). Those may affect the generalizability of the results. Although gender was not the primary focus of the research, unequal gender representation could influence how resilience, meaning in life, and stress are experienced and reported, as gender differences in stress responses and coping strategies are well-documented. Future studies should aim for a more balanced gender distribution to ensure that findings are more representative and to explore potential gender differences in the moderating effects of resilience and meaning in life on perceived stress. Third, the data were collected from a single university, which restricts the generalizability of the findings to the broader Chinese population. Future research should sample from multiple universities or diverse settings to better understand how these variables function across different cultural contexts and educational environments. Last, perceived stress is measured by a two-item scale as long questionnaires often lead to negative moods, fatigue, and unwillingness to continue. while empirically supported for global perceived stress assessment, they may overlook nuanced dimensions of stress reactivity and coping. Future studies incorporating multidimensional scales (e.g., 10-item PSS) could enhance granularity.

## 5. Conclusions

The analysis of the research data has led to several important conclusions. First, consideration of future consequences (CFCs) among college students is a significant predictor of perceived stress. Individuals with a future-oriented mindset tend to experience lower levels of stress. Additionally, resilience acts as a mediator in the relationship between CFCs and perceived stress, with college students who exhibit high resilience reporting lower stress levels. Furthermore, a strong sense of meaning in life moderates the relationship between CFCs and perceived stress, although the effect of low meaning in life is not statistically significant. These findings highlight the critical importance of enhancing resilience and fostering a sense of meaning in life to reduce perceived stress among college students. Therefore, initiatives aimed at increasing resilience and promoting meaningful life experiences should be prioritized in college settings, as such approaches are likely to effectively mitigate perceived stress.

## Figures and Tables

**Table 1:** Descriptive statistics and Pearson’s correlations.

Variable	*M*	*SD*	1	2	3	4	5	6
CFC	3.159	0.446	1.000					
resilience	3.373	0.600	0.175 ***	1.000				
PS	2.609	0.889	−0.144 ***	−0.316 ***	1.000			
MIL	4.749	0.859	0.282 ***	0.479 ***	−0.141 ***	1.000		
MIL-P	4.482	0.997	0.241 ***	0.432 ***	−0.187 ***	0.873 ***	1.000	
MIL-S	5.015	0.977	0.250 ***	0.402 ***	−0.057	0.867 ***	0.515 ***	1.000

Notes: *** *p* < 0.001.

**Table 2:** Tests of the mediation effect of resilience in consideration of future consequence and perceived stress.

Regression Equation		Overall Fit Index		Regression Coefficient				
Outcome Variable	Predictor Variable	*R* ^2^	*F*	*B*	*SE*	*LLCI*	*ULCI*	*t*
PS	CFC	0.021	20.486	−0.144	0.032	−0.206	−0.082	-***
resilience	CFC	0.033	11.012	0.175	0.032	0.113	0.237	5.520 ***
	AGE			0.021	0.017	−0.127	0.055	1.221
	GENDER			−0.080	0.071	0.256	−0.219	−1.137
PS	CFC	0.131	36.432	−0.089	0.031	−0.148	−0.029	−2.905 **
	resilience			−0.296	0.031	−0.356	−0.236	−9.708 ***
	AGE			−0.075	0.016	−0.107	−0.044	−4.646 ***
	GENDER			−0.062	0.067	−0.194	0.069	−0.931

Notes: ** *p* < 0.01; *** *p* < 0.001.

**Table 3:** Tests of the moderation effect of meaning in life in consideration of future consequences and perceived stress.

Regression Equation		Overall Fit Index		Regression Coefficient				
Outcome Variable	Predictor Variable	*R* ^2^	*F*	*B*	*SE*	*LLCI*	*ULCI*	*t*
PS	CFC	0.114	31.177	−0.088	0.032	−0.151	−0.025	−2.748 **
	MIL			0.036	0.036	−0.034	0.106	1.013
	CFC*MIL			−0.062	0.026	−0.112	−0.011	−2.410 *

Notes: * *p* < 0.05; ** *p* < 0.01.

**Table 4:** Conditional indirect effect of consideration of future consequences and perceived stress via resilience at different levels of meaning in life.

Moderator		Effect	*Boot SE*	*Boot LLCI*	*Boot ULCI*
MLQ	(−1SD)	−0.026	0.044	−0.112	0.060
MLQ	Mean	−0.088	0.032	−0.151	−0.025
MLQ	(+1SD)	−0.149	0.038	−0.223	−0.075

Notes: Bootstrapping sample = 5000.

**Table 5:** Tests of the moderation effect of search for meaning in consideration of future consequences and perceived stress.

Regression Equation		Overall Fit Index		Regression Coefficient				
Outcome Variable	Predictor Variable	*R^2^*	*F*	*B*	*SE*	*LLCI*	*ULCI*	*t*
PS	CFC	0.122	33.461	−0.098	0.032	−0.161	−0.036	−3.096 ***
	MIL-S			0.100	0.034	0.034	0.166	2.960 ***
	CFC*MIL-S			−0.059	0.027	−0.112	−0.007	−2.213 *

Notes: * *p* < 0.05; *** *p* < 0.001.

**Table 6:** Tests of the moderation effect of presence of meaning in consideration of future consequences and perceived stress.

Regression Equation		Overall Fit Index		Regression Coefficient				
Outcome Variable	Predictor Variable	R^2^	F	B	SE	LLCI	ULCI	*t*
PS	CFC	0.112	30.566	−0.079	0.032	−0.141	−0.017	−2.502 *
	MIL-P			−0.045	0.034	−0.112	0.022	−1.307
	CFC*MIL-P			−0.043	0.024	−0.091	0.004	−1.780

Notes: * *p* < 0.05.

**Table 7:** Conditional indirect effect of consideration of future consequences and perceived stress via resilience at different levels of search for meaning.

Moderator		Effect	*Boot SE*	*Boot LLCI*	*Boot ULCI*
MLQ-S	(−1SD)	−0.039	0.045	−0.128	0.050
MLQ-S	Mean	−0.098	0.032	−0.161	−0.036
MLQ-S	(+1SD)	−0.158	0.038	−0.232	−0.084

Notes: Bootstrapping sample = 5000.

## Data Availability

The data presented in this study are openly available in the Harvard Dataverse at doi:10.7910/DVN/LZLHXP.
